# Effects of TiAl Alloy as a Binder on Cubic Boron Nitride Composites

**DOI:** 10.3390/ma14216335

**Published:** 2021-10-23

**Authors:** Yuxi Liu, Wei Zhang, Yingbo Peng, Guojiang Fan, Bin Liu

**Affiliations:** State Key Laboratory of Powder Metallurgy, Central South University, Changsha 410083, China; yuxiliu@csu.edu.cn (Y.L.); ybpengnj@njau.edu.cn (Y.P.); guojiang.fan@gmail.com (G.F.); binliu@csu.edu.cn (B.L.)

**Keywords:** cubic boron nitride, TiAl alloy, high-temperature and high-pressure sintering method, flexural strength

## Abstract

Owing to their extreme hardness, cubic boron nitride (cBN) composites are widely used in cutting applications. The performance of cBN composites is closely related to the characteristics of the binder. Therefore, novel binders must be developed to improve the performance of cBN composites. In the present work, TiAl intermetallics were used as binders to fabricate cBN composites by employing a high-temperature and high-pressure sintering method. The phase transformation, sintering reaction mechanism, thermal stability, and mechanical properties of the resultant cBN composites were investigated. It was found that during the sintering process, Ti atoms preferentially reacted with boron nitride particles, whereas Al atoms enriched and transformed into TiAl_3_ phases and formed cBN/AlN, AlB_2_/TiN, and TiB_2_/TiAl_3_-layered structures eventually. The composites maintained good oxidation resistance at 1200 °C. A decrease in the particle size of the TiAl binder improved the uniformity of particle size distribution and increased the flexural strength of the composites.

## 1. Introduction

Cubic boron nitride (cBN) has excellent hardness, wear resistance, thermal stability, and chemical inertness. In order to manufacture polycrystalline cubic boron nitride (PcBN), binders are generally used [1,2]. Traditional PcBN binders mainly include metallic binders (titanium, aluminum, cobalt, and their alloys), ceramic binders (nitrides, carbides, and silicides), and cermet binders, represented by MAX phases, where M is an early transition metal, A is an A-group element (mostly IIIA and IVA), and X is either C and/or N [3,4]. However, these binders have some serious disadvantages. For example, Al atoms can react with cBN to form AlB_2_ and AlN. Moreover, residual Al atoms cause the thermo-softening of PcBN. The addition of Ti converts AlB_2_ into harder AlN and TiB_2_ phases; however, the combustion reaction of Ti and Al commonly transforms cBN into hBN due to the local high temperature. When MAX phases, such as Ti_3_AlC_2_ and Ti_3_SiC_2_, are used to sinter PcBN, they decompose under high temperatures and high pressure. The formation of reaction products is difficult to control in this case [5,6,7,8]. Therefore, a binder that takes advantages of both metallic and ceramic binders is a potential material to substitute for traditional binders for superior PcBN performance.

Intermetallic compounds generally consist of both ionic and covalent bonds; thus, they possess the characteristics of metals and ceramics at the same time. Intermetallic compounds are the best potential high-temperature materials between superalloys and ceramics. γ-TiAl, a lightweight, high-temperature intermetallic compound, has the advantages of low density, high specific strength, superb corrosion resistance, high-temperature resistance, and excellent oxidation and fatigue resistance. Thus, it is widely applied in the aerospace and automotive industries [9,10,11]. In comparison to other intermetallic compounds, γ-TiAl has a higher oxidation resistance, a higher specific melting point, and superior high-temperature stability. Hence, it is considered as a potential PcBN binder.

TiAl alloy has not been adopted as a PcBN binder so far; however, an intermetallic compound of Ti and Al is produced during the sintering process of PcBN [12,13]. The use of the pre-alloyed TiAl intermetallic compound as a PcBN binder has the following advantages: (1) although TiAl is intrinsically brittle at room temperature, it has enough strength and toughness at high temperatures to avoid thermo-softening. (2) TiAl can react with cBN to generate high-strength TiB_2_, TiN, AlB_2_, and AlN phases, which can adequately bond cBN particles [14]. (3) The combustion reaction of Ti and Al powder particles does not occur during the sintering process, thereby preventing the local temperature from rising to form hBN. (4) Solid-phase sintering makes the distributions of Al and Ti more uniform and the sintering structure more consistent. (5) TiAl can maintain the intermetallic structure during the sintering process and form a matrix-reaction product-intermetallic compound, which help to retain the advantages of using a metallic binder for a complete bonding. This process can avoid the presence of metal residues and maintain good mechanical properties at high temperatures. Moreover, the distribution of reaction products is controllable during this process. It should be noted that TiAl is easily oxidized during the high-temperature sintering. Thus, the oxygen level needs to be strictly controlled during the entire process. Solid-phase sintering also requires particle uniformity; otherwise, a large number of pores without binder filling exist, which act as crack-propagation channels.

The current study presents the results of using high-temperature and high-pressure sintering to prepare PcBN composites by using TiAl as a binder. The type and distribution of sintered products were clarified. The sintering mechanism and the effects of the grain sizes of binder particles on the uniformity and controllability of PcBN structures were systematically investigated.

## 2. Materials and Methods

cBN composites were prepared by combining pre-alloyed Ti-48Al-2Cr-2Nb powder (purity > 99%, −150 mesh) and cBN powder (purity > 99%, <10 μm). Stainless steel tanks and balls were used. The TiAl powder was weighed with a ball-to-powder ratio of 15:1, placed in a ball mill under the protection of argon for dry milling (at 200 r/min for 15 h and at 250 r/min for 15 h and 20 h) and in ethanol for wet milling (at 250 r/min for 15 h and 300 r/min for 25 h), and mixed with the cBN powder for six hours to ensure uniformity. The TiAl powder initial weight ratio was 10%. The phase compositions of the TiAl powder before and after ball milling were detected by X-ray diffraction (XRD) The mixed powder was sintered at 1400 °C under a pressure of 5 GPa for 30 min to obtain a cBN composite sample.

The sintered samples were then cut and polished by a diamond grinding disc. The densities of the composite samples were measured by Archimedes’ principle. The phase compositions of the synthesized samples were analyzed by an XRD (Max255Ovb+, RigakuD, Tokyo, Japan) under Cu-Kα radiation. The microscopic morphologies of the samples were revealed by a scanning electron microscope (SEM; Nova NanoSEM230, FEI, Hillsboro, OR, USA, VEGA3-SBH, TESCAN, Brno, Czech Republic) equipped with an energy-dispersive spectroscope (EDS). Raman spectroscopy (LabRAM HR800, Horiba Jobin Yvon, Paris, France) was performed to analyze bonding characteristics between different atoms. Elemental distributions were analyzed by an electron probe micro-analyzer (EPMA; Jxa-8530F, JEOL, Tokyo, Japan). The thermal analysis of the samples was performed by a synchronous thermal analyzer (STA 449 F3 Jupiter, NETZSCH, Selb, Germany), and their flexural strengths were measured by an mechanical testing machine (Instron 3369, Boston, MA, USA).

## 3. Results

### 3.1. Phase and Microstructure

**Figure 1:**
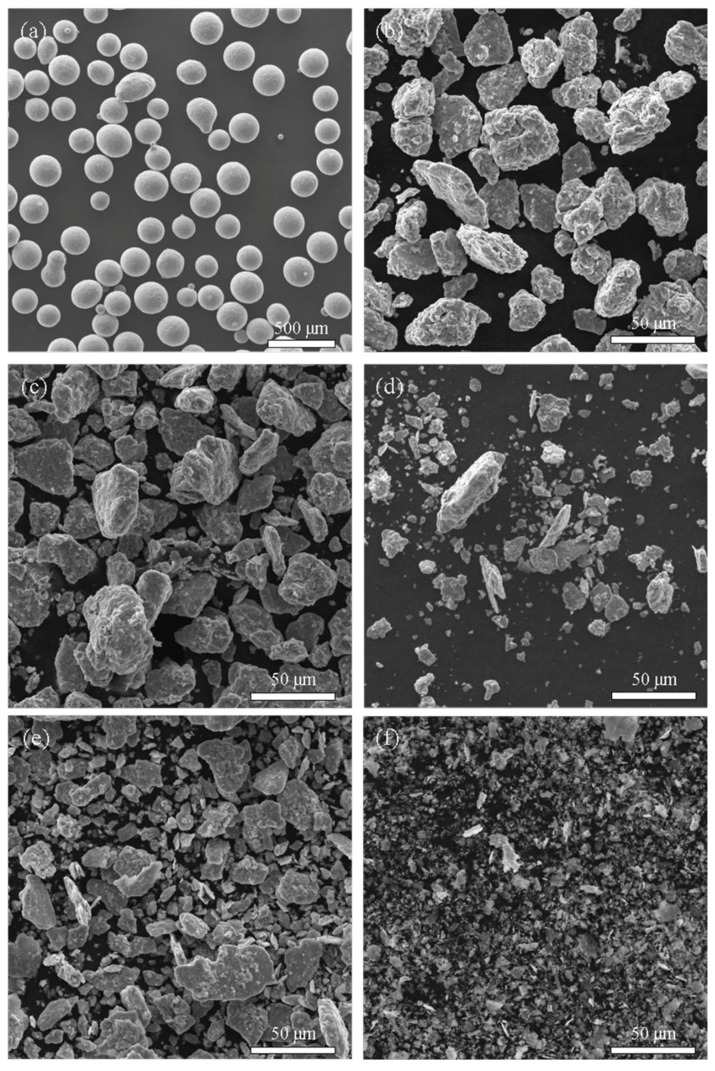
Surface morphologies of TiAl powder: (**a**) primary powder, (**b**) ball-milled powder at 200 r/min for 15 h (particle size = 29 μm), (**c**) ball-milled powder at 250 r/min for 15 h (particle size = 26 μm), (**d**) ball-milled powder at 250 r/min for 20 h (particle size = 17 μm), (**e**) ball-milled powder at 250 r/min for 15 h (particle size = 15 μm), (**f**) ball-milled powder at 300 r/min for 25 h (particle size = 5 μm). (**b**–**d**) dry-milled, (**e**,**f**) wet-milled.

**Figure 2:**
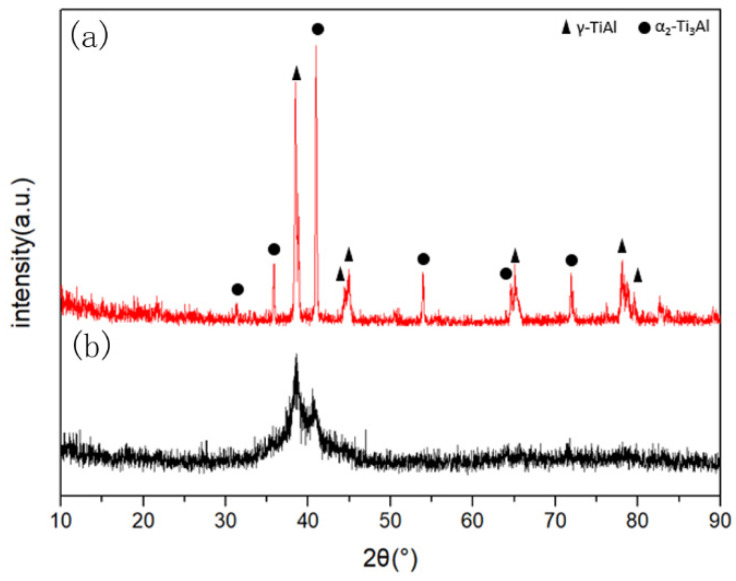
XRD spectra of TiAl powder: (**a**) primary powder, (**b**) ball-milled powder for 15 h (particle size = 26 μm).

It is difficult for commercial TiAl powder to be used as a binder due to larger particle size (particle size of general binders is less than 10 μm); ball milling was used to crush the TiAl powder to facilitate sintering. The particle morphology of the TiAl powder after ball milling is displayed in Figure 1, and the corresponding XRD pattern is presented in Figure 2. Figure 1b–d describe the dry-milled TiAl powder, while Figure 1e,f show the wet-milled powder. TiAl powder particles were spherical before ball milling, and the powders after ball milling were composed of rough-surface and irregular blocks or granular particles. Owing to the cold welding of the dry-milled powder, some of the finer debris agglomerated, and some coarse and massive particles possessed a multilayered structure. The wet-milled powders were ruptured into fine flakes. The corresponding full width at half maximum of the XRD peak increased. 

**Figure 3:**
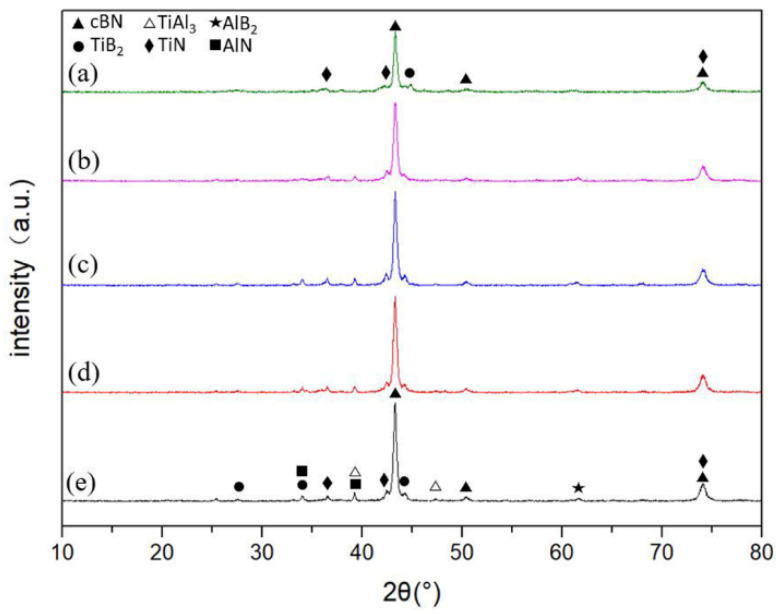
XRD spectra of sintered cBN composites: (**a**) 5 μm, (**b**) 15 μm, (**c**) 17 μm, (**d**) 26 μm, (**e**) 29 μm.

The relative density of the sintered cBN composites reached 99%. Their XRD patterns are exhibited in Figure 3. The phase structures of the sintered cBN composites were composed of a majority of cBN phases, as well as TiN, TiB_2_, AlN, AlB_2_, and TiAl_3_ phases. However, TiAl and Ti_3_Al phases that existed in the original TiAl powder were not observed in the sintered cBN composites. As the particle size of the binder decreased, the XRD peaks gradually broadened, and the TiAl_3_ phase disappeared eventually.

**Figure 4:**
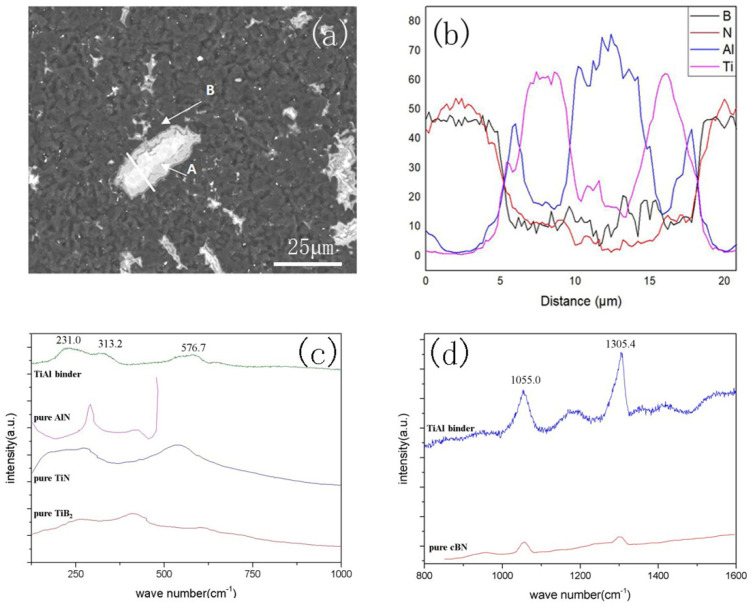
Microstructure and phase analysis of cBN composites: (**a**) 15 μm, (**b**) EDS of the line in (**a**), (**c**) Raman spectra of point A in (**a**), (**d**) Raman spectra of point B in (**a**).

To clarify the morphology and composition of the binder after sintering, the binder-rich region was examined by the SEM. Figure 4a presents the SEM images of the cBN composites, with an average binder particle size of 15 μm. In the microstructures, original white and gray TiAl regions were distributed within the black cBN matrix. An Al-rich white area appeared at the center of the original TiAl region, and a Ti-rich gray reaction–diffusion layer existed between the white area and the cBN matrix. A very thin Al-rich layer appeared between the diffusion layer and the cBN matrix.

A Raman spectroscopy analysis was performed on points A and B in Figure 4a, and the corresponding results are presented in Figure 4c,d, respectively. At point A of the diffusion layer, the peaks at 231.0 cm^−1^ [15], 313.2 cm^−1^ [16], and 576.7 cm^−1^ [17] appeared from TiB_2_, AlN, and TiN, respectively. At point B, the peaks at 1055.0 cm^−1^ and 1305.4 cm^−1^ [18] originated from cBN. The main bonding in the composite sample were Ti-B and Al-N. The peaks of Ti-rich compounds were much higher than those of Al-rich compounds, indicating that the content of nitrides and borides in Ti was much higher than in Al.

**Figure 5:**
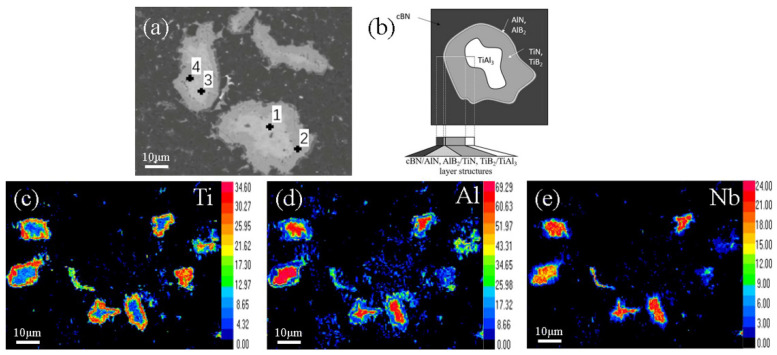
Microzonal composition analysis and EPMA results of cBN composites: (**a**) microzonal composition analysis; (**b**) layer structure of cBN composite; (**c**) EPMA map of Ti element; (**d**) EPMA map of Al element; (**e**) EPMA map of Nb element.

An EPMA analysis was performed on each point in Figure 5a. The corresponding results are presented in Table 1. The white phases surrounded by the diffusion layer are indicated by 1# and 3# (Figure 5a). In these areas, the content of Al was high, whereas the contents of B and N were low, indicating that these areas were mainly composed of TiAl_3_. The phases of the gray diffusion layer are indicated by 2# and 4# (Figure 5a). The content of Ti in these areas was significantly higher than in other regions. The contents of B and N were also high in the diffusion layer. Thus, it is concluded that Ti was rapidly consumed to form a large portion of the TiB_2_ and TiN phases. Figure 5e shows that the Nb elements were distributed in the center of the original TiAl area and hardly diffused to the matrix. There was no phase that contained Nb in the XRD spectra, which means that Nb was solubilized in the TiAl_3_ phase. The line scan in Figure 4b indicates that when the EDS signal moved from the matrix to the original TiAl region, B and N content decreased, whereas Al and Ti content increased. Al content reached the first peak ahead of Ti, and then the Al content gradually decreased, while the Ti content maximized. The existence of AlN and AlB_2_ peaks in the XRD spectra and the presence of Al-N peaks in the Raman spectrum indicate that the nitrides and borides of Al were distributed in the thin Al-rich region against the black matrix and formed cBN/AlN, AlB_2_/TiN, and TiB_2_/TiAl_3_-layered structures, as showed in Figure 5b.

### 3.2. Sintering Mechanism

The bonding mechanism of TiAl and cBN was compared with that between cBN and traditional binders. Li et al. [19] sintered cBN-Al in the high-temperature range of 800–1400 °C at 5 GPa. It was found that Al reacted with cBN to form AlN at 900 °C, and free B atoms diffused to form AlB_2_.

(1) BN+Al→AlN+[B]

(2) $$2\left[B\right]+\mathrm{Al}\to {\mathrm{AlB}}_{2}$$

Rong et al. [20,21] studied the microstructure of cBN-Al by TEM and reported that the relative content of cBN had an important effect on the reaction pathway. When the cBN content ranged between 80 and 90%, AlB_12_ was formed. Al atoms first filled the pores between cBN grains and then reacted with cBN grain surfaces to form AlN. B atoms, which were released by BN, diffused through the AlN layer and reacted with unreacted Al to form AlB_2_ and α-AlB_12_. When TiN was added, AlB_2_ with poor properties reacted with TiN to form harder AlN and TiB_2_ phases. Finally, cBN reacted with AlN, TiB_2_, and TiN phases to form a three-dimensional network matrix, which effectively improved the bonding between the binder and the cBN grains.

(3) $${\mathrm{AlB}}_{2}+\mathrm{TiN}\to \mathrm{AlN}+{\mathrm{TiB}}_{2}$$

(4) $$2\mathrm{BN}+\mathrm{TiN}+3\mathrm{Al}\to 3\mathrm{AlN}+{\mathrm{TiB}}_{2}$$

Deng [22] asserted that Al could react aggressively with Ti to form intermetallic compounds, which can generate excessive heat within a very short period time. This abrupt heating process reversely transformed a part of cBN into hBN. Active Al and Ti atoms in liquid TiAl, TiAl_3_, and Ti_3_Al reacted with cBN. hBN was gradually converted to cBN, with AlN as a catalyst.

When the MAX phase of Ti_3_AlC_2_ is used as a binder and the content of cBN is low, Ti_3_AlC_2_ decomposes very minimally [6].

(5) $${\mathrm{Ti}}_{3}{\mathrm{AlC}}_{2}\to {\mathrm{Ti}}_{3}C_{2}+\left[\mathrm{Al}\right]$$

The formation of TiC can isolate cBN and Ti_3_AlC_2_, which prevents further reactions between them. A larger amount of cBN increases the contact area between the substrate and Ti_3_AlC_2_. The following reaction continues to occur until the transition layer fully grows.

(6) $${\mathrm{Ti}}_{3}C_{2}\to 2\mathrm{TiC}+\left[\mathrm{Ti}\right]$$

Free Ti and Al atoms react with cBN, and Ti_3_AlC_2_ is consumed continuously until it completely decomposes.

In the present study, after the high-temperature and high-pressure sintering, the composite was mainly composed of cBN and some newly formed ceramic phases (TiB_2_, AlN). The main reactions that occurred during the sintering process are presented below.

(7) $$3\mathrm{TiAl}+2\mathrm{BN}\to {\mathrm{TiB}}_{2}+2\mathrm{TiN}+3\left[\mathrm{Al}\right]$$

(8) $$3\left[\mathrm{Al}\right]+2\mathrm{BN}\to {\mathrm{AlB}}_{2}+2\mathrm{AlN}$$

It was not found that α-AlB_12_ was present in the final phase composition. Although α-AlB_12_ was more stable than AlB_2_ at high temperatures, α-AlB_12_ would appear when the molar content of Al was over 40%. In this work, the TiAl powder initial weight ratio was 10%, which was much less than 40%.

A thermodynamic analysis was performed to reveal the reactions of Ti and Al with the matrix.

(9) $$3\mathrm{Al}+2\mathrm{BN}\to {\mathrm{AlB}}_{2}+2\mathrm{AlN}$$

(10) $$3\mathrm{Ti}+2\mathrm{BN}\to {\mathrm{TiB}}_{2}+2\mathrm{TiN}$$

**Figure 6:**
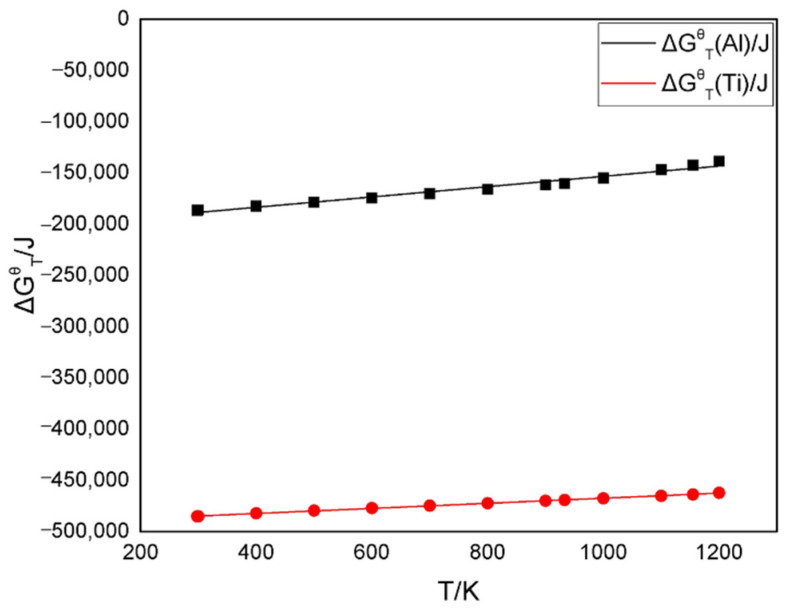
Fitting function curves of the Gibbs free energy.

The fitting free energy curves of reactions (9) and (10) are displayed in Figure 6 [23].

(11) $$\Delta G_{T}^{\theta }\left(\mathrm{Ti}\right)=-492,353+24.78T\left(298-1200K\right)$$

(12) $$\Delta G_{T}^{\theta }\left(\mathrm{Al}\right)=-203,740+50.35T\left(298-1200K\right)$$

When TiAl was bonded with cBN, the reaction between Ti and cBN was much more active than that between cBN and Al. Ti atoms in the lattice first reacted with cBN to form TiN and TiB_2_ and then attracted more Ti atoms to aggregate. Al atoms were continuously rearranged to both sides of the particle. Al atoms, which were located inside of the particle, were continuously enriched. A large amount of TiAl_3_ was replaced by Al atoms. Al atoms near the boundary reacted with cBN to further bond the particle.

During the heating process of sintering, Al first reached the melting point (660 °C). The molten Al penetrated cBN gaps, which wrapped and isolated Ti from the matrix to a certain extent and generated Al nitrides and borides. An AlN melt film was formed on the cBN surface to bond the matrix, resulting in the formation of PcBN with high density and good toughness [18,24]. The MAX phase first decomposed into Al atoms, which reacted with the matrix. A further decomposition produced Ti atoms during sintering. The reaction mechanism is presented in Table 2.

After the alloying of Ti and Al, the reaction order changed. A large amount of Ti nitrides and borides were selectively generated. The explosive combustion reaction of Ti and Al to cause cBN reversal into hBN was avoided.

### 3.3. Properties Analysis

**Figure 7:**
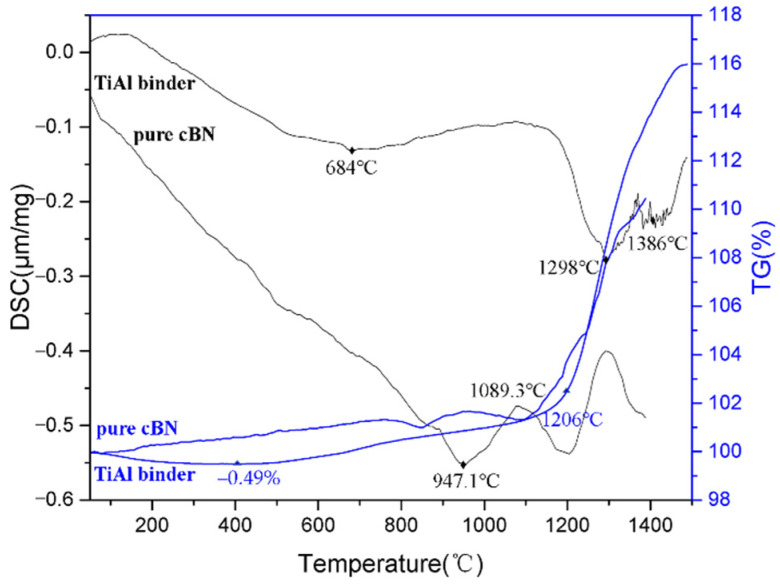
TG-DSC curves of the cBN composite (15 μm) and pure cBN abrasives.

A thermal analysis was performed on the cBN composite, with an average TiAl particle size of 15 μm. Its TG-DSC (Thermo Gravimetry–Differential Scanning Calorimetry analysis) curve and pure cBN abrasives are displayed in Figure 7. The –0.49% mass loss in the early stage of the TG curve occurred due to the desorption of the adsorbed gas in the composite during the heating stage. The sample was then slowly oxidized to a temperature of 1206 °C, and subsequently, the oxidation process accelerated. At this time, the oxidation and evaporation of the sample proceeded simultaneously. The weight gain during oxidation was much greater than that during evaporation. The maximum weight loss occurred at 1298 °C. The surface oxide film began to break, and a large amount of oxygen entered the interior of the composite and quickly reacted with cBN. Finally, a large amount of substrate reacted at 1386 °C. Compared with pure cBN abrasives, the sample had less weight gain at a temperature of nearly 1250 °C [25].

**Figure 8:**
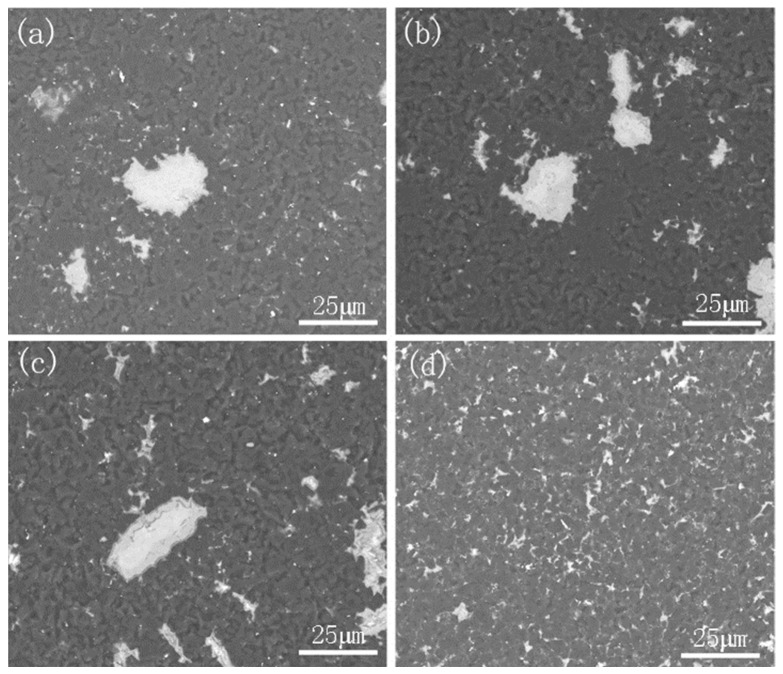
Backscattered electron images of cBN composites: (**a**) 29 μm, (**b**) 17 μm, (**c**) 15 μm, (**d**) 5 μm.

The flexural strength of the cBN composites with different TiAl particle sizes are presented in Table 3. It is clear that as the TiAl particle size decreased, the flexural strength increased significantly. Different ball milling methods also caused a change in flexural strength. The oxygen content in the wet-milled samples increased significantly, resulting in a decrease in the flexural strength. The highest flexural strength was obtained when the TiAl particle size was 5 μm. The SEM images of the cBN composite with different particle size are displayed in Figure 8.

**Figure 9:**
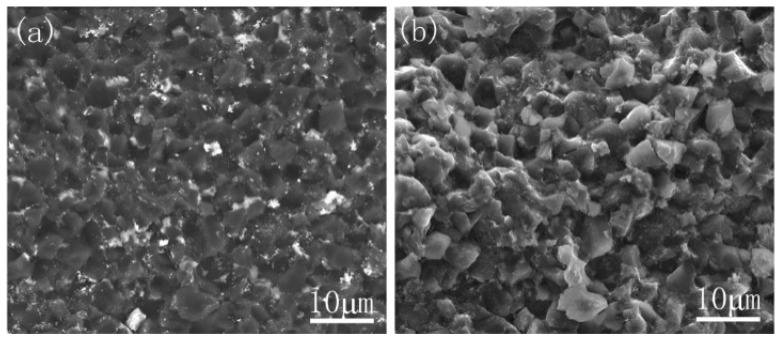
Three-point bending fracture of the cBN composite (5 μm): (**a**) BSED and (**b**) ETD.

The fracture morphology of the three-point-bending samples of the cBN, with an average TiAl particle size of 5 μm, is displayed in Figure 9. The cBN matrix had a typical rock sugar-like intercrystalline fracture morphology, and the crystal interface at the position of the binder was not obvious. The AlN phase was observed at gray cBN grain boundaries, and the TiB_2_ ceramic phase was observed in the diffusion layer. At the same time, as TiAl can still maintain a certain strength at a relatively high temperature, it could avoid thermo-softening caused by metal residues. However, as TiAl alloy has a higher melting point than Al, when the particle size was large, the gaps between cBN particles could not be completely filled. These unfilled gaps had a certain effect on the initiation and propagation of cracks, thereby affecting the toughness and density of the composite.

To further illustrate the influences of binder particle size and grain size on the flexural strength of the composite, the XRD patterns of the TiAl powder before and after ball milling (Figure 2) were peak fitted. The grain size (D) was calculated by the Scherrer formula.

(13) $$D=\frac{K\lambda }{B\cos\theta }$$

where K = Scherrer constant (0.89), θ = Bragg diffraction angle, λ = wavelength, and B = full width at half maximum.

The average grain size of the TiAl powder before and after ball milling were calculated as 41.83 nm and 6.10 nm, respectively. Therefore, the grain size was significantly reduced after ball milling. It can be inferred that when the ball-milled TiAl powder was used to bond cBN, its fine grain size could strengthen the sintered cBN composite. Meanwhile, the reduction in the particle size further strengthened the composite due to the dispersion strengthening by making the distribution of binder particles more uniform. These two strengthening effects improved the flexural strength of the cBN composite.

## 4. Conclusions

(1) In the microstructure of the cBN composite prepared by adopting γ-TiAl alloy as the binder, the TiAl_3_, TiN and TiB_2_-phase diffusion layers were distributed on the cBN matrix. A thin layer of AlN and AlB_2_ was distributed at the cBN grain boundary, forming cBN/AlN, AlB_2_/TiN, and TiB_2_/TiAl_3_-layered structures. The density of the composite reached 99% after sintering.

(2) Ti atoms reacted with cBN continuously. During the reaction, Al atoms were continuously rearranged to both sides of the interface reaction layer by forming AlN and AlB_2_ phases, which were located at the boundary, while the TiAl_3_ phase appeared on the inner side.

(3) The particle size and oxygen content of TiAl significantly affected the mechanical properties of the cBN composite. The grain size of the powder after ball milling was reduced from 41.83 nm to 6.10 nm, leading to an increase in the strength of the cBN composite, owing to the fine-grain strengthening mechanism. The distribution of the binder was more uniform due to the decrease in its particle size. When the particle size decreased from 29 μm, the flexural strength of the composite further increased. The composite prepared from TiAl, with an average particle size of 5 μm, had a peak flexural strength of 360 MPa.

(4) TiAl avoided the inverse conversion of cBN to hBN caused by the local heating of the combustion reaction, which retained part of the unreacted intermetallic compound binder. However, the preparation process before sintering significantly increased the oxygen content, which led to the oxidation of TiAl during the sintering process.

## Figures and Tables

**Table 1:** Microzonal composition analysis of cBN composites in Figure 5.

Point	Ti	Al	B	N	Nb
1	11.67	53.10	19.20	3.47	12.56
2	27.18	15.49	31.60	24.18	1.55
3	12.13	67.29	0.00	6.22	14.35
4	32.14	8.92	22.43	34.90	1.60

**Table 2:** Reaction mechanisms of several binders.

Binder Type	Preferred Element	Sintering Type	Local Heating
Ti + Al	Al	liquid-state	√
Ti_3_AlC_2_	Al	solid-state	×
TiAl	Ti	solid-state	×

**Table 3:** Flexural strengths of cBN composites.

NO.	TiAl Particle Size/μm	Ball Milling Method	Flexural Strength/MPa
1	29	dry	99
2	26	dry	101
3	17	dry	182
4	15	wet	170
5	5	wet	360

## Data Availability

The data presented in this study are available upon request from the corresponding author.
